# A Bioinformatics Study of Immune Infiltration-Associated Genes in Sciatica

**DOI:** 10.1155/2022/7372431

**Published:** 2022-03-24

**Authors:** Tao Ma, Guanhua Li, Yuheng Ma, Zhaoqi Ren, Houyun Xie, Chaoyong Sun, Lei Tian, Hao Zhang, Wei Wang

**Affiliations:** ^1^Department of Anesthesiology, PLA Rocket Force Characteristic Medical Center, Beijing, China; ^2^Department of Infusion, PLA Rocket Force Characteristic Medical Center, Beijing, China

## Abstract

Sciatica has been widely studied, but the association of sciatica with immune infiltration has not been studied. We aimed to screen key genes and to further investigate the impact of immune infiltration in patients with sciatica. The bioinformatics analyzes were performed based on the GSE150408 dataset. Subsequently, we used CIBERSORT to study the immune infiltration in the disease group. Results showed that 13 genes were with differentially expressions in the sciatica group compared to healthy participants, including 8 up-regulated and 5 down-regulated genes. Through the LASSO model and SVM-RFE analysis, a total of 6 genes have intersections, namely SLED1, CHRNB3, BEGAIN, SPTBN2, HRASLS2, and OSR2. The ROC curve area also confirmed the reliability of this method. CIBERPORT analysis showed that T cell gamma delta infiltration decreased and neutrophil infiltration increased in the disease group. Then the association of these six key genes with immune infiltration was further verified. We found six overlapping genes and found that they were closely associated with the total immune infiltration in the sciatic nerve disease group. These findings may provide new ideas for the diagnosis and therapeutics of patients with sciatica.

## 1. Introduction

Sciatica is commonly caused by lumbar disc herniation involving peripheral neuropathy [1, 2]. According to statistics, the incidence of sciatica in one's life is as high as 40%. The common treatment methods for sciatica include nonsurgical conservative treatment and nonsurgical treatment. 90% of acute sciatica can be effectively relieved by nonsurgical treatment [3].

Proteomic analysis has identified proteins related to sciatica or intervertebral disc degeneration, which may be involved in the pathophysiological process of sciatica [4]. It is generally believed that mechanical compression combined with immunity and inflammation can lead to sciatica during lumbar disc herniation. Many cytokines related to immunity and inflammation are activated in lumbar disc herniation [5, 6].

In this study, we used two machine learning methods to explore and identify the key genes of patients with sciatica and preliminarily analyzed the immune cell infiltration. Then further evaluate the correlation between immune cell infiltration and the central gene in sciatica so as to provide new research ideas for the treatment and early detection of sciatica.

## 2. Materials and Methods

### 2.1. Screening of Differentially Expressed Genes (DEGs)

We downloaded GSE150408 in the GEO database (https://www.ncbi.nlm.nih.gov/geo/). The platform of the GSE150408 mRNA microarray is GPL21185, which was used for the following analyzes.

### 2.2. Identification of Feature Gene

The feature genes were screened by two machine learning algorithms, least absolute convergence and selection operator (LASSO) and support vector machine-recursive feature elimination (SVM-RFE) and validated in the validation dataset. Machine learning is a new type of algorithm analysis tool. This study used the method of machine learning to identify features for algorithm analysis. Two machine learning algorithms, LASSO and SVM-RFE, were applied for marker screening. LASSO is a regression algorithm regularized through “*glmnet*” package in R. SVM-RFE is a supervised learning technique that can rank features based on recursion. We adopted the “e1071” package to complete the SVM algorithm.

### 2.3. Analyzes of Immune Infiltration

The CIBERSORT deconvolution algorithm was adopted for the estimation of different immune cell proportions. Totally, we obtained twenty-two types of immune cells. CIBERSORT filters data with *p* < 0.05. We then calculated each immune cell type's percentage and displayed it as a bar graph. The “pheatmap” package was adopted for the construction of the heat map of the twenty-two types of immune cells. Comparisons of levels of the twenty-two types of immune cells were done using a package.

### 2.4. Statistical Analysis

Analyzes of the association of immune cells with feature genes were performed using Spearman's rank via *R* software. We used the “ggplot2” package for the visualization of the plot. *P* < 0.05 indicated statistically significant.

## 3. Result

### 3.1. Diagnostic Feature Biomarkers Screening

**Figure 1:**
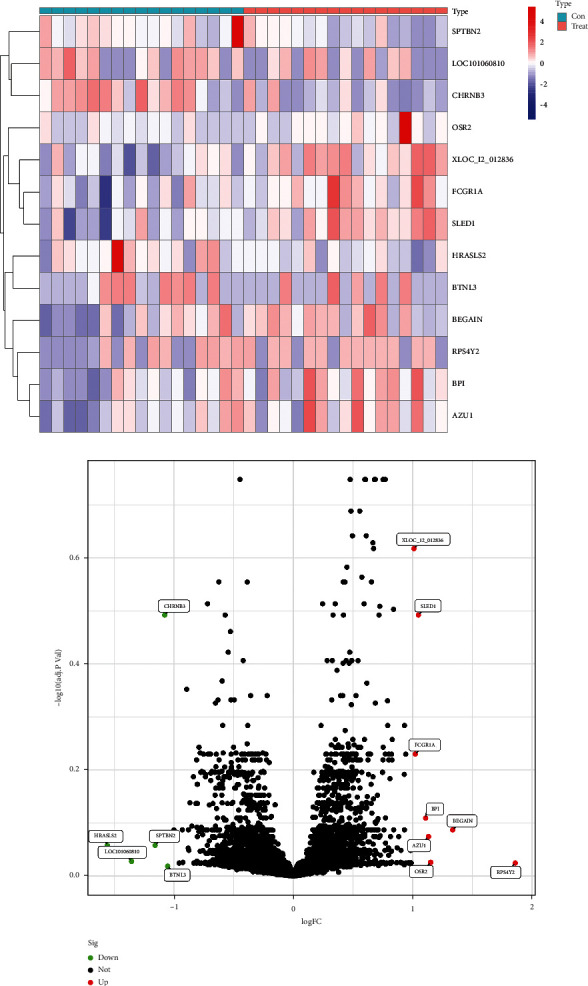
Differential analyzes based on the datasets: (a) heat map of DEGs (adjust *P* < 0.05, |logFC| >1) and (b) the volcano map of DEGs.

**Figure 2:**
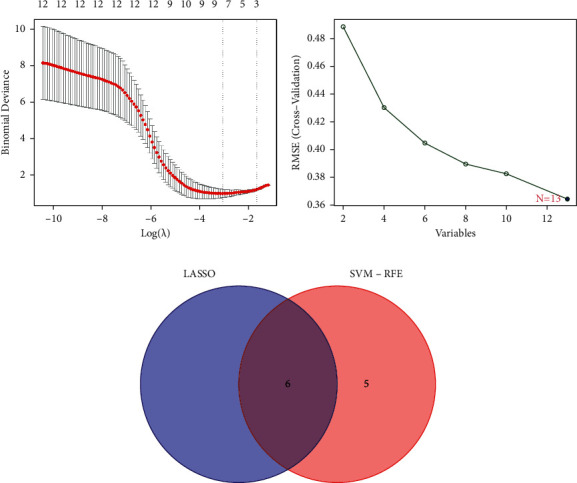
Establishment of prognostic genes: (a) selection of tuning feature in LASSO models, (b) selection of biomarkers using SVM-RFE and (c) the overlapped feature genes between 2 methods.

**Figure 3:**
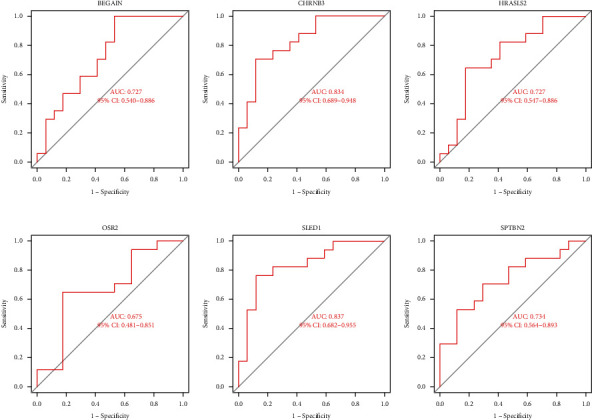
Diagnosis efficiency ROC curve of the feature genes: (a)–(f) ROC curve of SLED1 (a), CHRNB3 (b), BEGAIN (c), SPTBN2 (d), HRASLS2 (e), and OSR2 (f).

After removing the batch effects, thirteen DEGs were screened out: 8 significantly up-regulated and 5 significantly down-regulated (Figures 1(a) and 1(b)). Using the LASSO regression algorithm, we found 8 potential Figure 1(b) variables for the disease group (Figure 2(a)). A total of thirteen features were determined in Figure 2(b). SLED1, CHRNB3, BEGAIN, SPTBN2, HRASLS, and OSR2 were finally selected in Figure 2(c). Then, ROC was performed for the evaluation of the value of the prediction of the 6 characteristic genes. The AUCs for all 6 genes were greater than 0.8 (Figure 3(a)). It showed that the characteristic biomarkers have a high diagnostic ability, Figure 3(f).

### 3.2. Analyzes of Immune Infiltration

**Figure 4:**
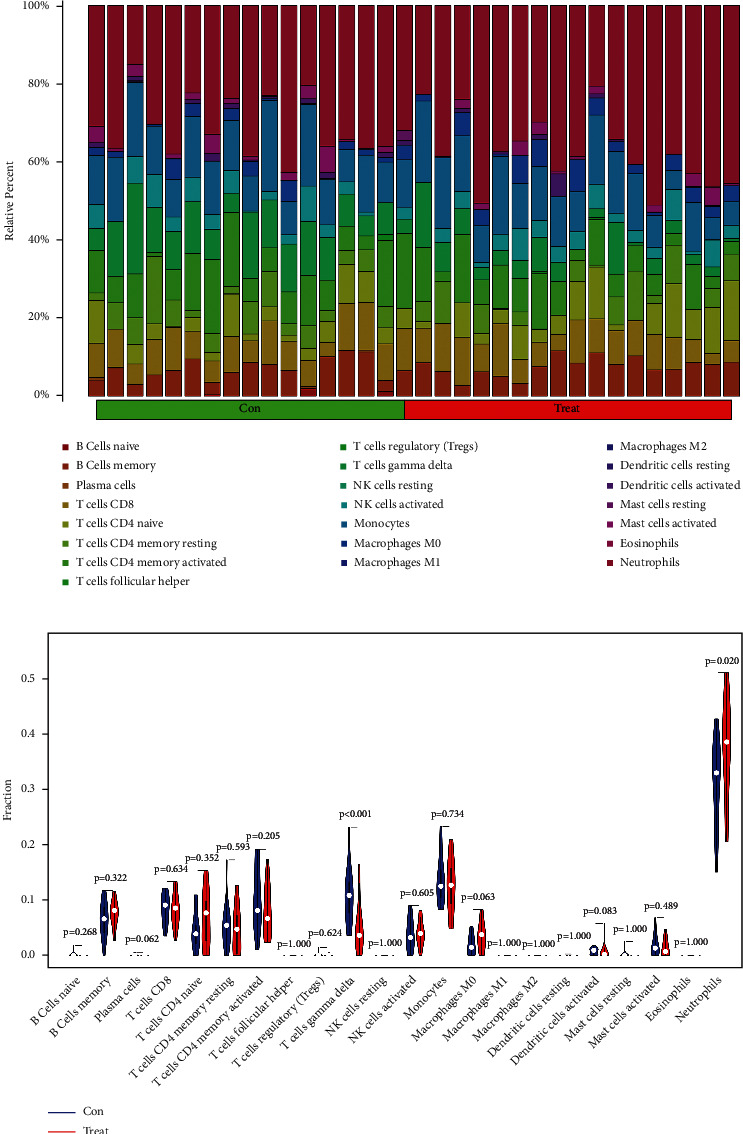
Visualization and evaluation of immune cell infiltration: (a) relative percentage of 22 kinds of immune cells, and (b) immune cells' violin plot image.

Immune infiltration in control and sciatica groups was explored with the twenty-two subpopulations of immune cells. The percentage of the twenty-two types of immune cells was visually displayed in Figure 4(a). CIBERPORT analysis showed that T cells gamma delta infiltration decreased and the degree of neutrophils infiltration increased in the sciatica group (Figure 4(b)).

### 3.3. Relationship of Central Genes with Immune Cells

**Figure 5:**
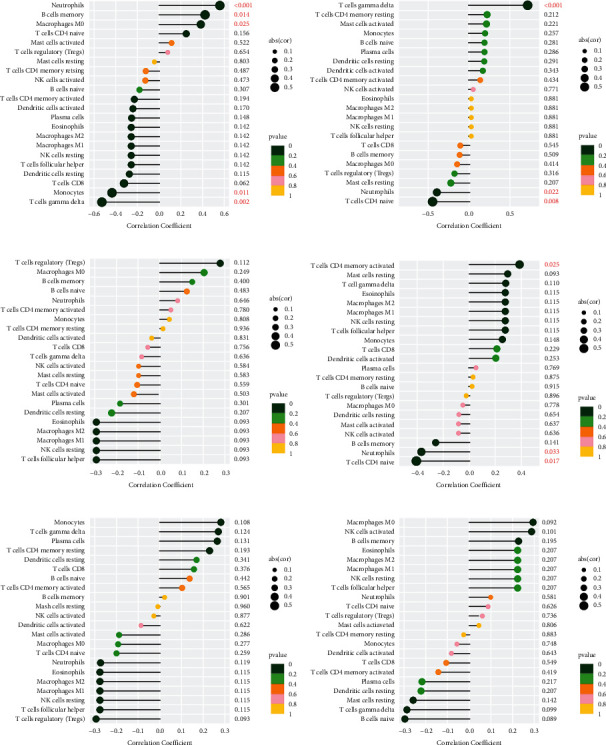
Correlation analyzes between immune cells and SLED1, CHRNB3, BEGAIN, SPTBN2, HRASLS2, and OSR2. (a) Association of SLED1 with immune cells. (b) Association of CHRNB3 with immune cells. (c) Association of BEGAIN with immune cells. (d) Association of SPTBN2 with immune cells. (e) Association of HRASLS2 with immune cells. (f) Association of OSR2 with immune cells.

As shown in Figure 5(a), SLED1 was related to macrophages M0 (*R* = 0.38, *P*=0.025), B cells memory (*R* = 0.42, *P*=0.014), neutrophils (*R* = 0.56, *P*=0.00073) positively and correlated with monocytes (*R* = −0.43, *P*=0.011), T cells gamma delta (*R* = −0.53, *P*=0.0016) negatively. CHRNB3 was positively associated with T cells gamma delta (*R* = 0.72, *P*=3.8*e* − 06) and negatively associated with neutrophils (*R* = −0.39, *P*=0.022), T cells CD4 naive (*R* = −0.45, *P*=0.0081) (Figure 5(b)).

SPTBN2 had a positive correlation with CD4 memory-activated T cells (*R* = 0.38, *P*=0.025) and a negative correlation with neutrophils (*R* = −0.37, *P*=0.033), T cells (CD4 naïve) (*R* = −0.41, *P*=0.017) (Figure 5(d)). CHRNB3 had a positive correlation with gamma delta-T cells (*R* = 0.72, *P*=3.8*e* − 06) and a negative correlation with neutrophils (*R* = −0.39, *P*=0.022). No correlation was found between BEGAIN, HRASLS2, OSR2, and immune cell infiltration (Figures 5(c)–5(f)).

## 4. Discussion

So far, there is no specific diagnostic method for sciatica. Combining medical history with a physical examination is the most common diagnostic method [7]. As a common clinical syndrome, sciatica is caused by two causes: one is internal and the other is external factors [8, 9]. When sciatica occurs, it often causes pain in the legs, back and below the knee, usually accompanied by tingling in the legs, numbness or muscle weakness [10, 11].

This study showed that 13 genes were differentially expressed in patients with sciatica. Through two methods, we identified six key genes, which are SLED1, CHRNB3, BEGAIN, SPTBN2, HRASLS2, and OSR2. We determined the association of these differentially expressed genes and immune infiltration in patients with sciatica. CIBERPORT analysis showed that T cell gamma delta infiltration decreased and neutrophil infiltration increased in the sciatica group. Up to now, there has still been a lack of research on brain-enriched guanylate kinase-associated protein. Studies have shown that brain-enriched guanylate kinase-associated protein participates in chronic pain. We demonstrated in the SNI model that mechanical abnormal pain, an abnormal pain condition caused by harmless stimuli, was significantly attenuated in BEGAIN deficient mice [12, 13]. Another key gene found is SPTBN2. It is the research on SPTBN2. At present, it is mainly used in research on congenital cerebellar ataxia and various cancers. In the study of cancer, miR-424-5p was found to be able to accelerate the development of endometrial cancer through regulating SPTBN2 and then the cldn4/PI3K/Akt axis [14–16]. Combined with bioinformatics and cell experiments, SPTBN2 may become a novel target of lung adenocarcinoma. SPTBN2, highly expressed in LUAD, might indicate poor prognosis. Cell experiments confirmed that SPTBN2 could promote the proliferative, migrative, and invasive abilities of LUAD cells [17].

The researchers found that glia was significantly activated in the brains of patients who experienced chronic pain, indicating that immune cells can spread and maintain disease states, including neuropathic pain, through communication with neurons rather than being regarded as bystanders [18]. During nerve injury, neuronal activity will be activated, resulting in the recruitment of monocytes/macrophages (peripheral) to the injured site. At the same time, microglia will release inflammatory-related mediators after activation, resulting in neuronal sensitivity [19].

The signal molecules of the immune system are cytokines. An increase of proinflammatory cytokines is related to the existence of pain after nerve injury, while antiinflammatory cytokines are related to the down regulation of the immune system and the relief of neuropathic pain [20, 21]. Immune system activation has been shown to promote and increase neuropathic pain [22].

Immune cells play an important role in different pathophysiological processes in the state of neuropathic pain. It brings the pain field to different directions and provides opportunities for new methods for the treatment of chronic pain.

However, there are still some limitations to the present study. This is a purely bioinformatics study without further experiments for validation, which weakened the evidence level of our results. In the future, we will conduct in vivo and in vitro assays to further explore the exact effects of the abovementioned immune-related genes and the potential underlying mechanisms on sciatica.

## 5. Conclusions

In summary, we systematically discussed the functions of immune-related genes of sciatica and provided new ideas for new methods for the treatment of chronic pain.

## Data Availability

The datasets used and analyzed during the current study are available from the corresponding author on reasonable request.
